# Phosphate Excretion Differentiates the Amount of Nephroprotective Effect of Amino Acid Ketoanalogues Treatment with Low Protein Diet in Chronic Kidney Disease—A Retrospective Single-Center Cohort Study

**DOI:** 10.3390/jcm15103986

**Published:** 2026-05-21

**Authors:** Ewelina Jędrych, Arkadiusz Lubas, Julia Bryłowska, Magdalena Mirkowska, Stanisław Niemczyk

**Affiliations:** 1 Department of Internal Diseases, Nephrology and Dialysis, Military Institute of Medicine—National Research Institute, 04-141 Warsaw, Polandsniemczyk@wim.mil.pl (S.N.); 2 Faculty of Medicine, University of Warsaw, 02-089 Warsaw, Poland

**Keywords:** chronic kidney disease, amino acid ketoanalogues, low protein diet, phosphaturia

## Abstract

Chronic kidney disease (CKD) affects more than 10% of the world’s population, increasing the risk of cardiovascular disease and mortality. **Background**: Nephroprotective interventions can reduce the risk of end-stage renal disease, delay the time to dialysis, and prolong life. However, there is ongoing debate about the effectiveness of combining amino acid ketoanalogues (KAA) with a low-protein diet (LPD) to slow CKD progression. This study aimed to retrospectively analyze kidney function outcomes after a 6-month KAA+LPD regimen in patients with CKD. **Methods**: The analysis included results from 38 non-dialyzed patients (12 F, 26 M; age 64.0 ± 13.6 years) with stable CKD in stages G4 to G5, who followed LPD with KAA (Ketosteril, Fresenius Kabi) treatment as part of the Polish National Health Fund Ketosteril Drug Program. **Results**: No significant change in estimated glomerular filtration rate (eGFR) was observed during 6 months of KAA+LPD therapy. However, eGFR increased or decreased in half of the patients (*p* < 0.001), and this change was associated only with initial protein intake and urinary phosphate excretion. Initial high phosphate excretion was independently associated with an increase in eGFR, and initial phosphaturia > 0.5 g/24 h identified eGFR improvement (sensitivity 84.2%; specificity 57.9%; AUC 0.712; *p* = 0.018) in CKD patients who started KAA+LPD treatment. **Conclusions**: Six-month treatment with KAA+LPD may be associated with stabilization of kidney function in patients with CKD stages G4-G5. The individual effect of KAA+LPD on renal function may be related to the initial protein intake level and urinary phosphate excretion. Further studies are needed to validate these findings across larger patient populations with a broader spectrum of symptoms.

## 1. Introduction

Worldwide, more than 850 million people suffer from kidney diseases, and chronic kidney disease (CKD) alone affects more than 10% of the European population, and the prevalence of CKD rises considerably with age [1,2]. CKD is expected to become the fifth leading cause of death by 2040 globally [1]. The progressive nature of CKD results in significantly increased cardiovascular morbidity and mortality despite renal replacement therapy (RRT). Therefore, it is extremely important to slow the loss of kidney function and delay the onset of kidney failure. Nephroprotective interventions reduce the risk of end-stage renal disease, prolong the time to dialysis therapy, and extend life. However, in the initial stages of kidney dysfunction, the basic therapeutic strategy is to manage the underlying disease. Diabetes, hypertension, and glomerulonephritis are among the most common causes of chronic kidney failure, making proper treatment of these diseases key to preventing exacerbation of kidney failure [3].

According to the most recent guidelines, there are five pillars of pharmacological nephroprotection in CKD patients: effective antihyperglycemic treatment, sodium–glucose co-transporter type 2 inhibitors or a glucagon-like peptide-1 receptor agonist with or without glycemic control, antihypertensive therapy, use of pharmaceuticals that antagonize the renin–angiotensin–aldosterone system with or without antihypertensive therapy (angiotensin II receptor blockers, angiotensin-converting-enzyme inhibitors, finerenone), as well as sodium bicarbonate in patients with metabolic acidosis [4,5].

Importantly, in any pharmacological approach, appropriate lifestyle interventions—such as changing unhealthy dietary habits, maintaining a healthy body mass, quitting smoking, and engaging in regular physical activity—are fundamental to reducing the risk of developing CKD [5]. Kidney Disease: Improving Global Outcomes (KDIGO) recommendations for CKD in stage G3–G5 suggest maintaining a low-protein diet (LPD) with protein intake of 0.8 g/kg body weight per day, or even a very low-protein diet (VLPD) (0.3–0.4 g/kg body weight/d) supplemented with essential amino acid ketoanalogues (up to 0.6 g/kg body weight/d) [5].

The nephroprotective effects of renin-angiotensin system inhibitors (RASi) and SGLT2i are primarily manifested by reductions in intraglomerular pressure, proteinuria, inflammation, and fibrosis. In contrast, excessive protein intake increases intraglomerular pressure, leading to hyperfiltration, glomerulosclerosis, and tubulointerstitial damage. Restrictions on dietary protein intake enhance the nephroprotective potential of pharmacotherapy. The results of previous studies validate the use of LPD in patients with CKD as a method to slow down the disease progression and extend the time to renal replacement therapy [6]. Additional supplementation with amino acid ketoanalogues (KAA) prevents muscle mass loss and malnutrition in these patients [7,8]. In studies, LPD combined with KAA treatment proved safe and demonstrated the ability to limit the degradation of muscle proteins while fully reusing the recycled amino acids [6,7,8,9]. Supplementing a low-protein diet with KAA reduces the risk of protein deficiency without nitrogen overload [6,10]. A reduced supply of nitrogen compounds prevents high urea concentrations and uremic symptoms, which are a clear indication for initiating RRT. Research data suggest that LPD alone reduces the phosphate supply. At the same time, calcium salts of amino acid ketoanalogues further reduce serum phosphate concentrations by forming insoluble calcium phosphate complexes in the intestines [11]. Hyperphosphatemia increases mortality in patients with CKD [12]. On the other hand, lowering and normalizing phosphate concentrations decreases parathyroid hormone (PTH) production, thereby limiting the development of secondary hyperparathyroidism, which has a positive effect on bone turnover and reduces the risk of osteoporosis [12].

However, results from studies evaluating a low-protein diet supplemented with ketoanalogues remain inconsistent. Some data suggest that the effects of KAA+LPD therapy may be related to baseline kidney function and metabolic changes in protein and phosphate metabolism, whereas other studies indicate that the treatment effectiveness may depend on factors such as the administered dose of ketoanalogues or treatment adherence [11,13]. In contrast, some authors emphasize the role of baseline renal function, suggesting that the nephroprotective effect of KAA+LPD may be more strongly related to CKD stage than to dietary factors alone [9]. These differences indicate that distinct clinical and metabolic factors may influence the response to KAA+LPD therapy and help explain the variability in results reported in the literature.

The study aimed to retrospectively analyze kidney function outcomes in patients with chronic kidney disease after a 6-month regimen of amino acid ketoanalogues combined with a low-protein diet.

## 2. Materials and Methods

### 2.1. Patients

The analysis retrospectively included test results from enrolled RRT-naïve patients with stable chronic kidney disease (KDIGO stages 4 and 5) who had an eGFR decline of <2 mL/min/1.73 m^2^ over the past 6 months, adhered to LPD with protein intake of less than 0.8 g/kg body weight per day for at least 3 months, and had a BMI of 18–30 kg/m^2^—in accordance with the 2021 guidelines of the Polish National Health Fund for the Ketosteril (Fresenius Kabi, Bad Homburg vor der Höhe, Germany) Drug Programme. Test results for patients qualified for the Ketosteril Drug Programme at our center were obtained in anonymized form from the Polish National Health Fund database. Daily protein intake was assessed based on nPNA (normalized protein equivalent of total nitrogen appearance) [14].

$$\mathrm{nPNA}=\frac{\left(24\mathrm{-h}\;\mathrm{urinary}\;\mathrm{urea}\;\mathrm{nitrogen}\;\mathrm{excretion}\;\left[g/d\right]\right)+0.031\;\left[g\;N/\mathrm{kg}/d\right]\times \mathrm{body}\;\mathrm{mass}\;\left[\mathrm{kg}\right])\times 6.25)}{\mathrm{body}\;\mathrm{mass}\;\left[\mathrm{kg}\right]}$$

Exclusion criteria included hypercalcemia, glycated hemoglobin (HbA1c) > 7.5%, uPCR (urine protein-to-creatinine ratio) > 2.0 g/g, severe malnutrition with an SGA (Subjective Global Assessment) score of ‘C’, lack of adherence to the LPD, and initiation of RRT. During the Drug Program, patients took 4–8 tablets of Ketosteril 3 times a day with meals, while restricting protein intake to 0.4–0.6 g/kg body weight per day. Subsequent assessments were performed every 3 months and included: nPNA, serum creatinine, uric acid, and bicarbonate levels; alkaline phosphatase activity; eGFR using the MDRD (Modification of Diet in Renal Disease) formula; BMI; and urinary protein and phosphate excretion. The test results were obtained from the local module of the National Health Fund’s central database. The data remained anonymous and were included in a statistical analysis.

### 2.2. Statistical Analysis

Statistical analysis was performed using the Statistica package (TIBCO Software Inc., Greenwood Village, CO, USA) version 13.3. The test results are presented as means with standard deviations and medians with interquartile ranges (IQR). The change in variables (∆) during treatment was calculated as the absolute difference between the result at the 6th month and at therapy initiation (month 0). The variables were checked for normal distribution using the Shapiro–Wilk test. Differences between variables with a near-normal distribution were assessed using Student’s *t*-test for paired or independent variables; otherwise, the Wilcoxon or Mann–Whitney test was used, respectively. Differences among more than two variables were analyzed using one-way analysis of variance (ANOVA) or the Kruskal–Wallis test, depending on whether the data met the normality assumption. Power analysis for difference tests was used. The Spearman test was used for assessing correlations between variables. The relationship between the change in condition and the outcome was examined using univariable and multivariable stepwise backward logistic regression, with goodness-of-fit assessment and k-fold cross-validation. The cut-off value was estimated using ROC analysis with the Youden index. For each test, a two-sided *p*-value < 0.05 was considered statistically significant.

## 3. Results

The study enrolled 38 subjects (12 women and 26 men; aged 64.0 ± 13.6 years) with stable chronic kidney disease stages G4 to G5, who started a low-protein diet combined with Ketosteril supplementation, between 2021 and 2024. Because patients were enrolled progressively, all eligible subjects continued treatment for at least 6 months. Results for the investigated parameters at 3-month intervals are presented in Table 1.

In the investigated group, during a 6-month follow-up, no significant changes in renal function, urea concentration, or proteinuria were observed. The mean decrease in estimated glomerular filtration rate (eGFR) at the sixth month compared to baseline was not substantial (mean −0.47 ± 4.97 mL/min/1.73 m^2^; median 0.50; IQR 6.0; *p* = 0.567). However, BMI decreased significantly at the 6-month observation. In addition, alkaline phosphatase decreased immediately in the 3rd month, with a further substantial decline. Moreover, we observed a significant trend towards a decline in urine phosphate excretion, visible as early as the third month of treatment (0.53 ± 0.18 vs. 0.44 ± 0.17 g/24 h; *p* = 0.011).

The absolute change in eGFR over 6 months of observation was correlated only with initial nPNA (r = 0.391; *p* = 0.015) and phosphate excretion (r = 0.341; *p* = 0.036), but not with the change over time in these variables. Moreover ∆eGFR correlated only with ∆Creatinine (r = −0.931; *p* < 0.001), ∆Phosphates (r = −0.511; *p* = 0.001), and ∆Urea (r = −0.628; *p* < 0.001).

**Figure 1:**
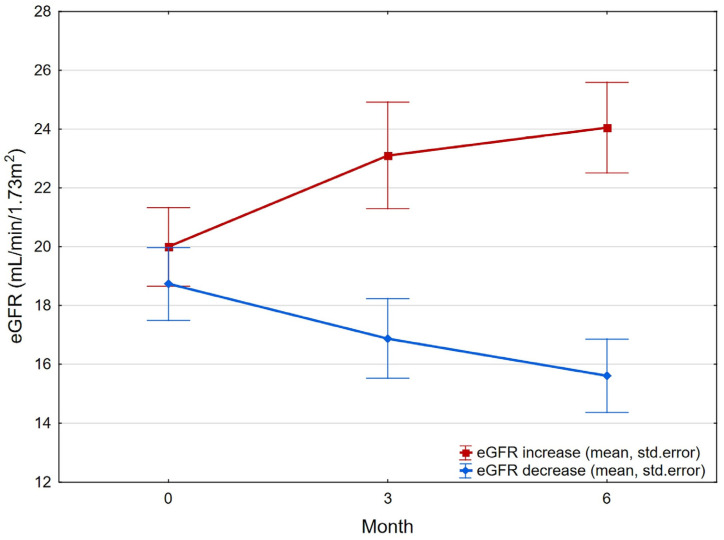
Graph of differential eGFR change during 6-month follow-up.

In 19/38 patients, eGFR increased at the 6th month by an average of 4.05 ± 4.43 mL/min/1.73 m^2^ (median 3.0, IQR 4.0). In the remaining group of patients, eGFR decreased by an average of 3.12 ± 2.03 mL/min/1.73 m^2^ (median 3.0, IQR 4.0), with a significant difference between increases and decreases in eGFR (*p* < 0.001; power 98.7%) (Figure 1).

Among all investigated baseline parameters, the groups divided by the change in eGFR differed only in nPNA and phosphate excretion, which were significantly higher in the group with an eGFR increase (nPNA 0.61 ± 0.15 vs. 0.51 ± 0.10 g/kg body weight/d; *p* = 0.019; phosphate excretion: 0.60 ± 0.21 vs. 0.45 ± 0.12 g/24 h; *p* = 0.027).

In the univariable logistic regression analysis, only nPNA and phosphate excretion were associated with the eGFR decrease. The stepwise backward multivariable logistic regression analysis included both variables and showed that only initial phosphate excretion independently predicted worsening of kidney function during 6-month LPD+KAA treatment (k-cross-validation: likelihood ratio = 6.881, *p* = 0.078; Hosmer-Lemeshow test = 7.774, *p* = 0.100; and Nagelkerke R^2^ = 0.22) (Table 2).

**Figure 2:**
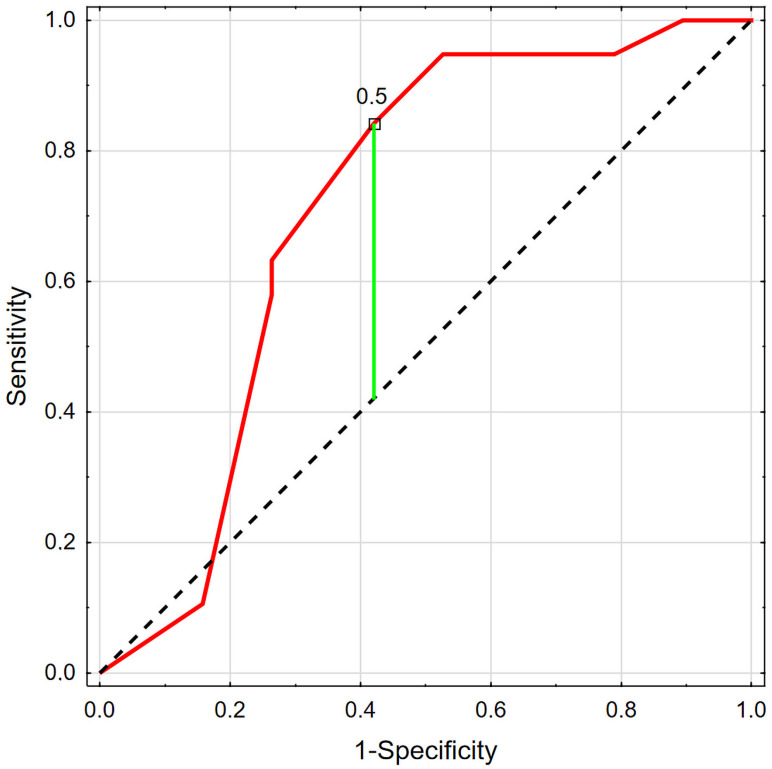
ROC analysis for initial phosphate excretion and GFR decrease during 6 months of follow-up. Red line—initial phosphate excretion; green line—Youden index; dashed line – reference threshold.

The ROC analysis (Figure 2) revealed that initial phosphate excretion ≤ 0.5 g/24 h could predict a decrease in eGFR over 6 months of KAA and LPD treatment, with a sensitivity of 84.2% and a specificity of 57.9% (AUC 0.712; *p* = 0.018). Patients with higher phosphate excretion (>0.5 g/24 h) had higher initial protein intake (0.651 ± 0.103 vs. 0.504 ± 0.117 g/kg body weight/d; *p* < 0.001; power 97.2%), whereas the groups did not differ in other investigated variables, including baseline eGFR.

As a categorical variable, phosphate excretion > 0.5 g/dL seems to be a strong and significant factor limiting the risk of GFR decline (OR = 7.33, 95%CI: 1.58, 33.97; *p* = 0.011).

At the inclusion, a weak correlation was found between male gender and Ketosteril dose (r = 0.342; *p* = 0.035), daily protein intake (r = 0.464; *p* = 0.003), creatinine (r = 0.321; *p* = 0.049), and uric acid (r = 0.329; *p* = 0.044) concentrations. Additionally, at baseline, Ketosteril dose showed a significant correlation with urea (r = 0.349; *p* = 0.032).

### Analysis of Treatment Outcomes According to eGFR

The study population was stratified by median estimated glomerular filtration rate (Group A: eGFR ≥ 19 mL/min/1.73 m^2^; n = 21; Group B: eGFR < 19 mL/min/1.73 m^2^; n = 17) to compare treatment outcomes depending on renal function. No significant change in renal function was observed during treatment in a comparative analysis of the study groups (Table 3). The mean change in renal function was 0.80 ± 5.2 mL/min/1.73 m^2^ after 6 months in subjects with higher baseline eGFR. However, in the group with lower baseline eGFR, the change in renal function was 0.05 ± 4.8 mL/min/1.73 m^2^ after 6 months (*p* = 0.556 for differences between groups).

## 4. Discussion

In this retrospective study, we showed that initiating KAA+LPD treatment can lead to different changes in eGFR over 6 months of observation. In half of the investigated patients with higher baseline protein intake and higher phosphate urinary excretion, initiation of KAA+LPD treatment was associated with an increase in eGFR, whereas in the remaining half, eGFR decreased. Although in our study, to be eligible for KAA, patients had to have proven adherence to a low-protein diet for at least 3 months, it ultimately turned out that the amount of daily protein intake, and especially the level of phosphaturia, significantly differentiated patients into those with an increase or decrease in eGFR during the 6-month observation. However, due to the small study group and significant limitations of the study, the presented results should be considered rather exploratory and hypothesis-generating, requiring external validation before clinical use.

Although an increasing number of studies show that CKD progression can be significantly slowed, reports concerning the effectiveness of KAA+LPD treatment on renal function improvement are inconclusive [3]. In a recent meta-analysis of 15 studies by Imam et al., which included 1596 patients treated with a very low-protein diet with the addition of nitrogen-free analogs or LPD only, the addition of KAA increased eGFR [15]. Moreover, in a one-year follow-up of a small group of nonagenarians, Annunziata et al. reported that the use of KAA together with LPD resulted in improved renal function (18.04 ± 1.31 vs. 24.30 ± 2.09 mL/min; *p* < 0.001) [16]. However, in another study by Ariyanopparut et al. that enrolled 1042 patients, with the median baseline eGFR of 31.1 mL/min/1.73 m^2^, the annual rate of decline in eGFR in patients receiving KAA+LPD was 4.5 (3.4–5.5) mL/min/1.73 m^2^ compared with 7.7 (6.0–9.4) mL/min/1.73 m^2^ in patients on LPD alone [17]. Although these studies showed that KAA+LPD may be beneficial for slowing the progression of renal function decline, they do not explain the differences in eGFR outcomes. Moreover, we found no similar reports in the literature demonstrating a relationship between the effectiveness of KAA+LPD treatment and baseline phosphaturia. Nevertheless, it seems reasonable that individuals with CKD who consume more protein could benefit more from protein restriction, because long-term elevated phosphaturia is an unfavorable prognostic factor for CKD [18]. Therefore, the trend toward reduced phosphaturia during KAA+LPD treatment, demonstrated in our study, is indicative of a nephroprotective effect that reduces the risk of CKD progression.

In multivariable logistic regression analysis, we showed that only initial phosphate excretion independently predicted worsening of kidney function. The initial phosphate excretion ≤ 0.5 g/24 h may result in a decrease in eGFR. Higher baseline phosphaturia may be caused by several factors. One of them can be higher dietary protein intake in this patient subgroup. Since phosphorus is mainly derived from protein-containing foods, increased protein consumption results in higher phosphate load and greater urinary phosphate excretion [19]. Dobronravov et al. demonstrated that higher dietary protein intake was associated with significantly higher fractional excretion of phosphate and 24 h urinary phosphate excretion, indicating that protein intake is an independent factor influencing urinary phosphate loss and contributing to disturbances of mineral metabolism in patients with renal dysfunction [20]. After the introduction of a low-protein diet supplemented with ketoanalogues, urinary phosphate excretion decreased in our patients, which may reflect a reduced dietary phosphate load rather than an improvement in renal function itself. Therefore, the higher baseline phosphaturia observed in the group with better eGFR response can be related to greater protein intake before treatment, and the subsequent decrease in phosphaturia may have resulted from dietary restriction of protein and phosphate, which could partially explain the apparent improvement in renal function parameters during follow-up. On the other hand, the higher initial phosphaturia may indicate better renal tubular function, which could be reflected in the observed increase in eGFR. Seiler et al. showed that fibroblast growth factor-23 (FGF-23) increases urinary phosphate excretion and decreases intestinal phosphate absorption in order to maintain phosphate homeostasis in chronic kidney disease [21]. The phosphaturic effect of FGF-23 requires functioning renal tubules, as it acts by reducing tubular phosphate reabsorption. Therefore, higher baseline phosphaturia may reflect better preserved tubular responsiveness to phosphaturic stimuli rather than merely higher dietary protein intake. In such patients, the remaining nephrons may still be capable of compensatory increase in phosphate excretion in response to higher protein load, which may explain the association between higher initial phosphaturia and more favorable short-term changes in eGFR observed in our study. Nevertheless, we did not measure the concentrations of parathyroid hormone and FGF-23. As emphasized by Provenzano et al., phosphaturia should be interpreted within a broader framework of prognostic and predictive biomarkers [22]. Patients with CKD have higher levels of tissue remodeling and inflammatory markers, such as MMPs. Specifically, MMP-2 correlates with serum phosphate, whereas MMP-8 and MMP-9 are linked to FGF-23 and proteinuria. Incorporating these biomarkers into clinical assessment may strengthen risk stratification, as they reflect the complex interplay among impaired mineral metabolism, chronic inflammation, and tissue remodeling in patients with chronic kidney disease. Conversely, in patients with a significant reduction in their previous protein intake, the improvement in eGFR calculated from creatinine may result from reduced creatinine production, misleadingly suggesting improved renal function, because creatine, the precursor of creatinine, is synthesized from amino acids such as glycine, arginine, and methionine, and its body pool depends on the dietary supply of these substrates [23]. Because creatine is continuously converted to creatinine, its pool must be replenished by the daily intake of essential amino acids derived from dietary protein. Therefore, patients with higher baseline protein intake may have had higher creatinine production, resulting in higher serum creatinine levels and lower estimated GFR at baseline. After the introduction of a low-protein diet supplemented with KAA, reduced protein intake could lead to decreased creatinine production, which in turn may increase eGFR calculated from creatinine without real improvement in glomerular filtration. Thus, the observed increase in eGFR in some patients may partially reflect changes in creatinine metabolism related to dietary protein restriction rather than recovery of kidney function. Although the relationship between protein intake and creatinine production, and, therefore, eGFR, is central to the presented results and affects the validity of the findings, the vast majority of studies evaluating the effects of low-protein diets have used eGFR calculated from creatinine. Therefore, our work significantly complements existing research by underscoring the importance of a previously underexplored aspect of phosphate excretion. Although our study is relevant to a wide range of analyses using creatinine-based eGFR, given the aforementioned limitations, the results should be interpreted as hypothesis-generating and should be verified in a larger group of subjects with additional assessment of renal function calculated from cystatin C [24]. On the other hand, in the Rizetto et al. study, three years of observation showed that LPD adherence was associated with an increase in creatinine-based eGFR, whereas LPD non-adherence was associated with a decline in kidney function [25]. As in our study, changes in creatinine-based eGFR could be interpreted as acute effects associated with decreased creatinine production due to initiation of LPD+KAA treatment. The same kidney function marker was adequate to monitor longitudinal changes in kidney function during chronic LPD adherence. This observation partially supports the correctness of our results, despite the lack of use of cystatin C-based eGFR.

In our observation, the eGFR improvement effect was not observed in patients with a low baseline protein intake. In some conditions, a reduction in eGFR in patients with low baseline phosphaturia may be associated with a poor renal prognosis due to malnutrition [26]. However, patients with severe malnutrition and an SGA score of ‘C’ were excluded from our study, which partially rules out this etiological factor.

Regarding the group of all investigated patients, the overall eGFR did not change significantly over the 6-month follow-up, and no relevant differences were observed in proteinuria or urea concentration. However, BMI, alkaline phosphatase activity, and urinary phosphate excretion decreased during treatment. Similar metabolic effects of a low-protein diet supplemented with ketoanalogues have been reported in previous studies. Li et al. demonstrated that in patients with eGFR < 18 mL/min/1.73 m^2^, a restricted protein diet with ketoanalogues significantly decreased serum phosphorus levels, increased calcium concentration, and reduced parathyroid hormone (PTH) levels [13]. Similar observations were reported by Satirapoj et al., who demonstrated that a very low-protein diet supplemented with ketoanalogues did not significantly improve eGFR, but prevented its decline compared with patients treated with protein restriction alone [27]. This suggests that the nephroprotective effect of ketoanalogues may be reflected by stabilization of renal function rather than its improvement. Taking into account all observed patients, our findings are consistent with this observation, as we also did not observe a significant change in overall eGFR despite favorable metabolic changes during therapy. In addition, Bellizzi et al. showed that urinary phosphate excretion decreased significantly only in patients receiving a very-low-protein diet supplemented with ketoanalogues (from 0.61 ± 0.23 to 0.34 ± 0.18 g/day at 6 months, *p* < 0.0001), whereas no significant change was observed in patients treated with a low-protein diet or free diet alone [28]. This observation is consistent with our findings, where urinary phosphate excretion also decreased during the 6-month follow-up. This effect may reflect reduced dietary phosphate load due to protein restriction, as well as the phosphate-binding effect of calcium salts contained in ketoanalogues, which may limit intestinal phosphate absorption and contribute to the nephroprotective effect of KAA+LPD therapy.

During the 6-month follow-up, we observed no significant difference in CKD progression between the study groups stratified by eGFR above or below 19 mL/min/1.73 m^2^. In contrast, a meta-analysis of 12 studies by Li et al. revealed that LPD combined with KAA significantly slowed down the decline in renal function in patients with eGFR > 18 mL/min/1.73 m^2^ (mean difference in eGFR = 5.81 mL/min/1.73 m^2^; *p* < 0.001) compared to patients who received a placebo instead of KAA [13]. On the other hand, the results of a randomized study by Garneata et al., which used inclusion and exclusion criteria similar to the ones in our study (stable stage G4 CKD defined as eGFR decrease < 4 mL/min/1.73 m^2^/year, uPCR < 1 g/g and no signs of malnutrition—SGA A/B), may be considered somewhat contradictory [9]. These authors found that VLPD supplemented with KAA significantly slowed down the progression of CKD in patients with baseline renal function of eGFR 20–30 mL/min/1.73 m^2^, and the most effective nephroprotective results were observed in patients with eGFR < 20 mL/min/1.73 m^2^ compared to patients on LPD without KAA. Despite conflicting reports in the above studies, likely due to differences in inclusion/exclusion criteria, our results suggest that baseline renal function in patients with CKD G4–G5 is less important for the effectiveness of KAA+LPD treatment than the amount of daily protein intake. Furthermore, a meta-analysis by Li et al. showed that very-low-protein diets do not confer a clear benefit in renal outcomes compared with low-protein diets, and adherence to VLPDs is significantly lower [13]. Based on these data, it can be concluded that KAA use may be more important for nephroprotection than a stricter low-protein diet.

We found a significant reduction in BMI from a slightly overweight mean to normal after 6 months of observation. In the study by Garneata et al., BMI, treated as a continuous variable, was a reliable predictor of the primary endpoint: a 50% increase in baseline GFR or the need to start dialysis (HR 0.01, *p* = 0.003) [9]. Although our study enrolled patients with a BMI ranging from 18 kg/m^2^ to 30 kg/m^2^, in a univariable logistic regression analysis, initial BMI was not associated with changes in eGFR during the first 6 months of follow-up. Our findings are consistent with those of other studies, in that the baseline BMI was not associated with any differences in renal function at baseline or at the end of follow-up [9,10]. Moreover, we observed a significant reduction in alkaline phosphatase as early as the third month of KAA+LPD treatment, which persisted through the follow-up period. This change was likely due to reduced phosphate intake (significantly lower phosphate excretion at 3 months) and a possible reduction in PTH and bone turnover, as a consequence of restricted protein intake and calcium supplementation along with the KAA treatment [13].

To sum up, our study demonstrates that treatment with amino acid ketoanalogues, combined with a low-protein diet, may be beneficial and preserve renal function at 6 months, regardless of baseline renal function. However, the magnitude of the nephroprotective effect during the first 6 months of KAA+LPD treatment may depend on baseline protein intake and phosphaturia.

Despite these interesting findings, our study has several limitations. First, a relatively small group of patients was enrolled, and the follow-up period was limited to 6 months. Therefore, it is unclear whether the results can be extrapolated to subsequent years of KAA supplementation. Furthermore, because patients were enrolled sequentially and the follow-up period was relatively short, we did not assess the long-term effects of KAA use. The study’s limitations can also be attributed to the relatively restrictive eligibility criteria, which limited population diversity and may have contributed to the lack of statistical significance in the treatment outcomes. Another important limitation is that only patients with stable renal function at baseline were eligible, and the treatment outcomes in these patients were not compared with those in a placebo control group, limiting the ability to determine the benefits of therapy. In addition, anonymized results precluded analysis of patients’ medications, and the retrospective design of the study prevented an assessment of factors influencing phosphate metabolism, which could otherwise alter the observed effects. Therefore, further studies should be conducted in a larger patient population with more diverse CKD symptoms, using less protein-dependent renal function markers to assess the effectiveness of a low-protein diet combined with amino acid ketoanalogues.

## 5. Conclusions

Amino acid ketoanalogues, combined with a low-protein diet, may be associated with stabilization of renal function in a selected group of patients with grade G4 to G5 chronic kidney disease, related to initial protein intake and the degree of phosphaturia, independent of baseline glomerular filtration rate. Further prospective studies involving larger groups of patients with chronic kidney disease are needed to confirm our results and identify the reasons for the observed associations.

## Figures and Tables

**Table 1:** Comparison of the results of the investigated variables at 3-month intervals.

Variable	Month 0	Month 3	Month 6	∆ (6–0)	*p*-Value	
	Mean ± SD	Mean ± SD	Mean ± SD	Mean ± SD	Kruskal–Wallis	According to Initial Data
	Median [IQR]	Median [IQR]	Median [IQR]	Median [IQR]		
Ketosteril (tabl.)	12.4 ± 1.1	12.4 ± 1.0	12.2 ± 1.8	−0.16 ± 1.48	0.71	0:3–0.715 ^#^
	12.0 [0.0]	12.0 [0.0]	12.0 [0.0]	0.00 [0.00]		0:6–0.463 ^#^
Albumin (g/dL)	4.4 ± 0.2	4.4 ± 0.2	4.3 ± 0.3	−0.06 ± 0.28	0.288	0:3–0.276 ^#^
	4.3 [0.2]	4.4 [0.3]	4.3 [0.3]	0.00 [0.30]		0:6–0.313 ^#^
BMI (kg/m^2^)	25.2 ± 3.0	25.2 ± 3.1	24.6 ± 2.8	−0.67 ± 1.85	0.519	0:3–0.879
	25.2 [5.4]	25.1 [4.4]	24.4 [4.0]	−0.05 [1.10]		0:6–0.033
eGFR (mL/min/1.73 m^2^)	19.4 ± 5.6	20.0 ± 7.6	19.8 ± 7.4	0.47 ± 4.97	0.999	0:3–0.409
	19.0 [7.0]	18.5 [13.0]	19.0 [12.0]	−0.50 [6.00]		0:6–0.567
Creatinine (mg/dL)	3.4 ± 1.0	3.4 ± 1.1	3.4 ± 1.1	0.03 ± 0.84	0.953	0:3–0.919
	3.3 [1.2]	3.4 [1.5]	3.5 [1.6]	0.00 [0.70]		0:6–0.806
Bicarbonate (mmol/L)	25.7 ± 4.4	25.1 ± 4.8	25.5 ± 4.3	−0.13 ± 2.57	0.717	0:3–0.350 ^#^
	27.0 [4.8]	24.8 [7.0]	25.4 [5.6]	−0.50 [2.80]		0:6–0.545 ^#^
Alkaline phosphatase (IU/mL)	89.8 ± 26.9	84.9 ± 29.5	85.0 ± 31.3	−4.87 ± 14.47	0.438	0:3–0.019 ^#^
	87.0 [32.0]	76.0 [39.0]	76.0 [40.0]	−7.00 [14.00]		0:6–0.005 ^#^
nPNA (g/kg body weight/d)	0.56 ± 0.13	0.53 ± 0.11	0.56 ± 0.13	0.00 ± 0.17	0.702	0:3–0.302
	0.56 [0.14]	0.54 [0.17]	0.56 [0.19]	−0.03 [0.25]		0:6–0.908
Proteinuria (g/24 h)	0.50 ± 0.47	0.54 ± 0.57	0.56 ± 0.51	0.05 ± 0.44	0.875	0:3–0.941 ^#^
	0.40 [0.56]	0.35 [0.71]	0.38 [0.85]	−0.01 [0.39]		0:6–0.763 ^#^
Calcium (mg/dL)	9.5 ± 0.5	9.5 ± 0.5	9.5 ± 0.6	−0.01 ± 0.55	0.972	0:3–0.822 ^#^
	9.6 [0.6]	9.6 [0.5]	9.6 [0.7]	0.00 [0.50]		0:6–0.776 ^#^
Potassium (mmol/L)	4.7 ± 0.6	4.6 ± 0.6	4.7 ± 0.5	−0.06 ± 0.54	0.581	0:3–0.133
	4.7 [0.6]	4.6 [0.8]	4.8 [0.7]	−0.05 [0.70]		0:6–0.472
Phosphates (mg/dL)	3.8 ± 0.8	3.7 ± 0.7	3.8 ± 0.7	0.04 ± 0.82	0.933	0:3–0.716
	3.7 [1.0]	3.6 [1.0]	3.8 [0.9]	0.10 [1.00]		0:6–0.754
Urea (mg/dL)	105.1 ± 38.9	104.9 ± 40.7	109.2 ± 36.8	4.12 ± 35.57	0.866	0:3–0.964
	98.0 [51.0]	100.0 [60.0]	109.0 [53.0]	−1.00 [52.00]		0:6–0.480
Uric acid (mg/dL)	6.5 ± 1.9	6.4 ± 1.8	6.1 ± 1.7	−0.37 ± 0.99	0.643	0:3–0.261 ^#^
	6.2 [2.4]	6.3 [2.2]	6.1 [2.1]	−0.25 [1.00]		0:6–0.052 ^#^
Glucose (mg/dL)	97.5 ± 13.6	92.1 ± 16.7	101.0 ± 30.4	3.45 ± 28.05	0.225	0:3–0.073 ^#^
	97.5 [14.0]	91.5 [20.0]	94.5 [19.0]	−0.50 [11.00]		0:6–0.850 ^#^
Phosphate excretion (g/24 h)	0.53 ± 0.18	0.44 ± 0.17	0.47 ± 0.18	−0.06 ± 0.22	0.072	0:3–0.011 ^#^
	0.50 [0.30]	0.40 [0.20]	0.40 [0.30]	−0.10 [1.00]		0:6–0.163 ^#^

BMI—body mass index; eGFR—estimated glomerular filtration rate; nPNA—normalized protein equivalent of total nitrogen appearance; ^#^—Wilcoxon test.

**Table 2:** Results of univariable and multivariable logistic regression analysis for the decrease in eGFR.

Variable	Univariable Analysis			Multivariable Analysis		
	OR	95%CI	*p*	OR	95%CI	*p*
Phosphate excretion (g/24 h)	0.004	0.000–0.428	0.020	0.004	0.000–0.428	0.020
nPNA (g/kg body weight/d)	0.001	0.000–0.518	0.029	-	-	-

CI—confidence interval; nPNA—normalized protein equivalent of total nitrogen appearance.

**Table 3:** Comparison of renal function in subjects grouped according to median eGFR.

Group	Month 0. Mean ± SD Median [IQR]	Month 6. Mean ± SD Median [IQR]	*p*-Value 0:6
Group A (n = 21) eGFR ≥ 19 mL/min/1.73 m^2^	23.19 ± 3.88 23.00 [5.00]	23.99 ± 6.54 24.00 [10.00]	0.518
Group B (n = 17) eGFR < 19 mL/min/1.73 m^2^	14.65 ± 3.20 16.0 [6.0]	14.71 ± 4.67 13.00 [7.00]	0.629

eGFR—estimated glomerular filtration rate.

## Data Availability

The datasets used and analyzed in this study are available from the corresponding author upon reasonable request.
